# Pulmonary hypertension optimal screening in interstitial lung disease (PHOS-ILD): a scoring system for the detection of pulmonary hypertension in patients with interstitial lung disease

**DOI:** 10.3389/fmed.2026.1908302

**Published:** 2026-10-05

**Authors:** Theodoros Karampitsakos, Evan Wasserman, Eleni Stagaki, Abhay Thottassery, Jorden Smith, Huda Asif, Brian Soto, Christian Arce Guzman, Jalen Hammonds, Anna Papasarantou, Agamemnon Bakakos, Ourania Papaioannou, Apostolos Matsioulas, Foteini Karakontaki, Konstantinos Loverdos, Eirini Kefalidou, Brenda M. Juan-Guardela, Jose D. Herazo-Maya, Adriano R. Tonelli, Debabrata Bandyopadhyay

**Affiliations:** 1 Division of Pulmonary, Critical Care and Sleep Medicine, Department of Internal Medicine, Ubben Center for Pulmonary Fibrosis Research, Morsani College of Medicine, University of South Florida, Tampa, FL, United States; 2 First Department of Pulmonary, Critical Care and Sleep Medicine, Hospital for Thoracic Diseases “SOTIRIA,” National and Kapodistrian University of Athens, Athens, Greece; 3 Division of Pulmonary and Critical Care Medicine, Integrated Hospital Care Institute, Cleveland Clinic, Cleveland, OH, United States

**Keywords:** identification, interstitial lung disease, pulmonary hypertension, scoring system, screening

## Abstract

**Background:**

Pulmonary hypertension (PH) is a common comorbidity of interstitial lung disease (ILD). Timely detection may improve outcomes through implementation of PH specific treatments.

**Methods:**

Retrospective, multicenter study aiming to identify features that predict PH in patients with ILD. In the derivation cohort (ILD cohort—University of South Florida), we identified predictors of PH in ILD and created a scoring system using the rounded value of odds ratio for each significant parameter. Correlation with hemodynamic parameters was investigated in the derivation and validation cohort (Idiopathic Pulmonary Fibrosis cohort—Cleveland Clinic).

**Results:**

We included 164 patients with ILD that underwent right heart catheterization (RHC) [derivation cohort: *n* = 118, median age (years, 95% CI): 69.0 (65.9–71.0), female gender: 65 (54.6%), median mean pulmonary arterial pressure (mPAP) (mmHg, 95% CI): 25.0 (23.0–27.0), median pulmonary vascular resistance (PVR) [Wood units (WU), 95% CI]: 3.6 (3.0–4.3), validation cohort: *n* = 46, median age (95% CI): 63.0 (60.0–66.0), female gender: 17 (37%), median mPAP (mmHg, 95% CI): 20.5 (18.0–27.2), median PVR (WU, 95% CI): 3.1 (2.2–3.4)]. Parameters and optimal cut-offs that significantly predicted precapillary PH in the derivation cohort were: age>42 years, heart rate recovery (HRR) at 1 min ≤ 15 bpm in 6-min walk test (6 MWT), 6-min walk distance (6 MWD) ≤ 270 meters, B-type natriuretic peptide (BNP) > 136 pg/mL, pulmonary artery to ascending aorta diameter ratio (PA/AA) > 1 in high-resolution computed tomography (HRCT), main PA diameter > 29 mm in HRCT, right ventricular systolic pressure > 42 mmHg on echocardiogram. We created a PH detection model that ranged from 0 to 28. The optimal cut-off threshold for the detection of PH was > 14 (80% sensitivity, 68% specificity, AUC: 0.78). A simplified scoring system without BNP and main PA diameter (range: 0–21) maintained the same AUC. The simplified scoring system exhibited positive correlation with mean pulmonary artery pressure both in the derivation (rho = 0.47, *p* < 0.0001) and validation cohort (rho = 0.36, *p* = 0.013).

**Conclusion:**

This study demonstrates that incorporation of demographic, functional, imaging, and echocardiographic characteristics can optimize the early identification of precapillary PH in patients with ILD, paving the way to structured screening strategies. Timely detection of PH might lead to timely implementation of emerging PH specific treatments in patients with ILDs.

## Introduction

Interstitial lung diseases (ILDs) represent a conglomerate of parenchymal lung diseases that primarily affect the interstitium, small airways and/or alveoli (1). The disease course of different ILDs is highly variable (2) and comorbidities have a major impact in the survival of these patients (3, 4). Pulmonary hypertension (PH) is a frequent comorbidity in patients with advanced ILDs (5). Variations in PH prevalence are observed across different ILD subtypes and severities (4, 6–8). A haemodynamic study of PH in the back then called chronic hypersensitivity pneumonitis showed that 44% of patients had precapillary PH (9). Prevalence might be lower in some other non-fibrotic ILDs, but seems to be high in Idiopathic Pulmonary Fibrosis (IPF), especially during end stage disease (4, 8, 10). In particular, PH has been reported in 14–17% of patients with early IPF (11–13), whereas among patients with IPF listed for lung transplantation the prevalence of PH was 74% based on the revised hemodynamic diagnostic criteria and 48% under the older hemodynamic criteria (14). The higher prevalence of PH in IPF than in other ILDs is plausible given the poorer prognosis of IPF (15).

Currently PH is defined as a mean pulmonary arterial pressure (mPAP) > 20 mmHg. Precapillary PH also includes in the definition a pulmonary arterial wedge pressure (PCWP) ≤ 15 mmHg and pulmonary vascular resistance (PVR) > 2 Wood units (WU) (16–18). ILD-PH is thought to arise from intersecting mechanisms including vascular endothelial dysfunction, immune dysregulation and alveolar hypoxia that drive pulmonary vasoconstriction and progressive vascular remodeling (19–21). As lung fibrosis advances, these vascular changes become more pronounced, leading to increasing functional impairment reflected in reduced 6-min walk distance (6 MWD), poorer quality of life and diminished survival (17, 21, 22). In terms of survival, ILD-PH has been associated with 73.3, 51.2, and 34.1%, 1-, 2-, and 3-year survival, respectively (17, 23, 24).

Despite its substantial clinical burden, the diagnosis of PH is often delayed, partly due to the considerable overlap between symptoms of ILD and PH (25). In fact, there appears to be an initial adaptive phase where PH is largely asymptomatic while symptoms appear later in the disease course with florid right heart failure. Early PH treatment, before the development of maladaptive remodeling, seems to lead to better outcomes, emphasizing the need for an early diagnosis to positively affect quality of life and survival (26, 27). This concept has been underscored by data from inhaled treprostinil suggesting that administration of the compound may improve outcomes (28–30). Therefore, there is a need for optimal and timely screening strategies (31). Current ESC/ERS guidelines recommend that patients with ILD and suspected PH undergo a comprehensive non-invasive assessment incorporating echocardiography, pulmonary function tests, arterial blood gases and chest computed tomography (CT) (17, 32). However, there is a pressing need for a validated, simple screening tool integrating routinely available clinical, functional and imaging variables to aid identification of patients at high risk of PH who should be referred for invasive haemodynamic assessment. The Pulmonary Hypertension Optimal Screening in Interstitial Lung Disease (PHOS-ILD) study was designed to address this need in patients with ILDs. The PHOS-ILD study, named after the Greek word “Phos,” which means light, aims to shed light on the optimal screening strategy in patients with ILD and suspected PH.

## Materials and methods

### Study design

The PHOS-ILD study is a multicenter, retrospective, non-interventional study aiming to identify parameters that could predict PH in patients with ILD. Consecutive patients with ILD that underwent right heart catheterization (RHC) for suspected PH were included in the analysis in the derivation cohort [ILD cohort—University of South Florida—Tampa General Hospital (USF/TGH)], between January 2022 and September 2023. Patients with post-capillary PH were excluded from the analysis. ILD was diagnosed following multidisciplinary discussion in accordance with Clinical Practice Guidelines (1, 33, 34). Suspicion of PH was defined by the presence of features suggestive of PH, according to a previously published algorithm developed by our group (4). Outcomes were selected based on published screening strategies for patients with ILD-PH (4, 35–37). Patients were divided into two groups (ILD-PH vs. ILD without PH). In each group, we recorded the proportion of patients exhibiting specific features, described in the literature as potentially predictive of PH. Subsequently, we compared the proportion of patients with available data exhibiting these features between these two groups and we calculated the corresponding odds ratios.

We used established cut-offs for parameters with established thresholds, i.e., ratio > 1 for pulmonary artery (PA) to ascending aorta (AA) diameter ratio (PA/AA) in high-resolution computed tomography (HRCT). Whereas in other parameters without established cut-offs we performed receiver operating characteristic (ROC) and split accordingly patients into two groups. A scoring system (PHOS-ILD score) was created using the rounded value of odds ratio for each parameter that was significant predictor of PH (4). ROC was used to identify the optimal cut-off threshold of the PHOS-ILD score for the detection of PH. Parameters that following removal did not worsen area under the curve (AUC) or parameters with substantial multicollinearity were excluded from the final score. Internal validation of the final PHOS-ILD score was performed using 2,000 bootstrap resamples and stratified 10-fold cross-validation. Bootstrap resampling was used to estimate optimism in the AUC. Calibration was assessed using cross-validated predicted probabilities from a logistic regression model incorporating the PHOS-ILD score, with calculation of the calibration intercept, calibration slope and Brier score. Moreover, we investigated whether there was a correlation of the PHOS-ILD score and parameters associated with PH diagnosis in RHC such as mPAP. Finally, we tested the performance of the PHOS-ILD score in an independent IPF cohort [Cleveland Clinic (CC)]. Study design is summarized in Figure 1A.

**FIGURE 1:**
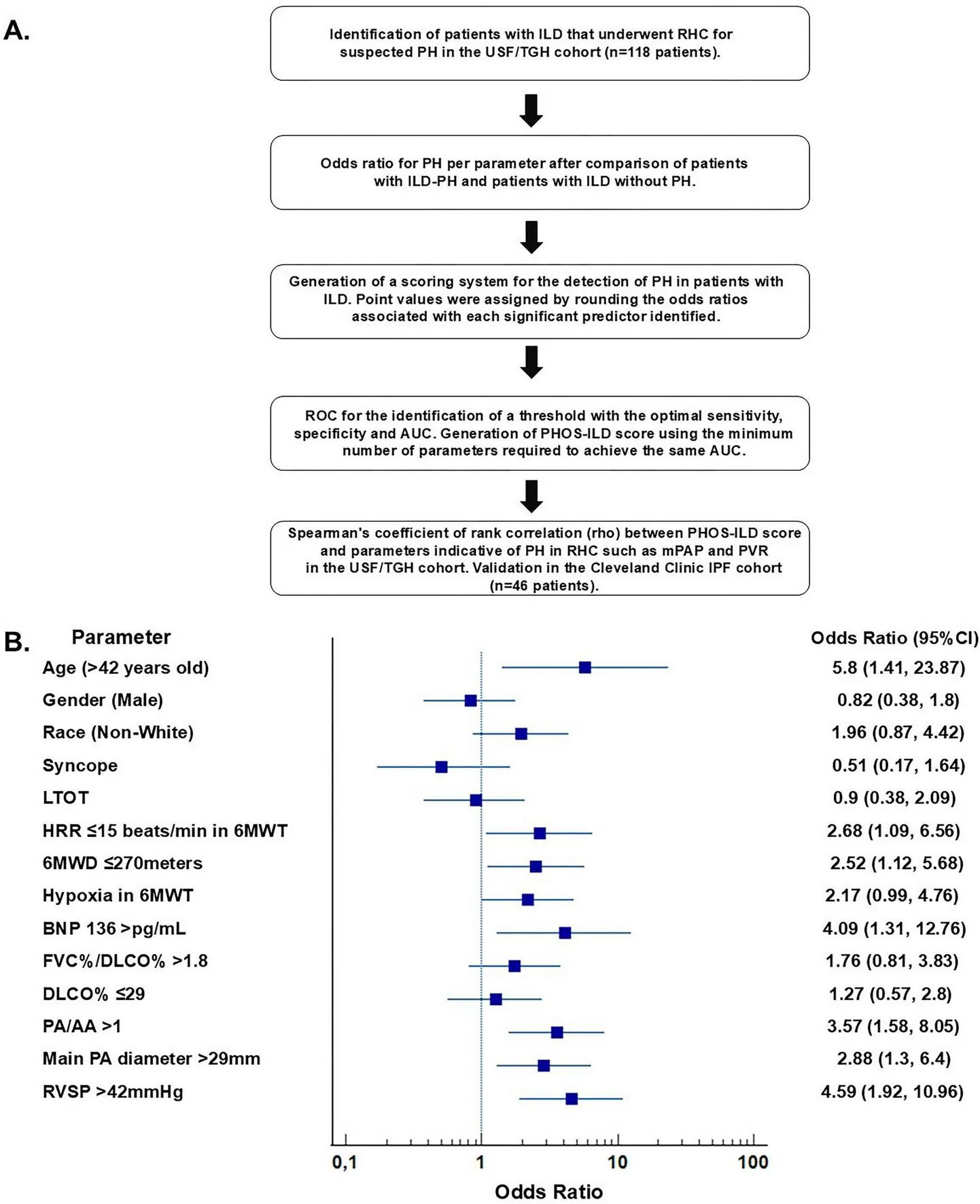
Schematic representation of the study design **(A)**. Odds ratio per parameter for PH in Tampa General Hospital/University of South Florida cohort **(B)**.

### Precapillary PH definition

All patients in this study underwent RHC. Precapillary PH was defined as mPAP > 20 mmHg with PCWP ≤ 15 mmHg and PVR > 2 WU (32). No PH was defined as a mPAP ≤ 20 mmHg.

### 6 -min walk test (6 MWT)

The procedure published in the INCREASE trial was followed in our study (28).

### Primary objective

The primary objective of the study was to identify significant predictors of PH in patients with ILDs and create a composite score based on these parameters to aid in the non-invasive screening of PH in ILD patients.

### Statistical analysis

Results are presented as mean ± standard deviation (SD) or median [95% Confidence Interval (CI)] as appropriate. To identify parameters significantly associated with the presence of PH in patients with ILDs, we calculated odds ratios and 95% CI in the derivation cohort. Subsequently, we used the rounded values of odds ratio for each significant predictor to develop a scoring system for the presence of PH in ILD named PHOS-ILD score. ROC was utilized to identify optimal thresholds for the detection of PH in ILD. In addition, we used Spearman’s coefficient of rank correlation to test the association between PHOS-ILD score and parameters associated with PH diagnosis, such as mPAP.

### Ethics approval

The study (STUDY007190) was approved by the Institutional Review Boards/Research Integrity and Compliance FWA No. 00001669/University of South Florida on 06/JUN/2024. All procedures performed in studies involving human participants were in accordance with the ethical standards of the institutional and/or national research committee and with the 1964 Helsinki Declaration and its later amendments or comparable ethical standards. Given the retrospective nature of the study, informed consent from patients was not needed. This has been approved by Institutional Review Boards of University of South Florida. Patients or the public were not involved in the design, or conduct, or reporting, or dissemination plans of our research.

## Results

### Demographics and baseline characteristics

We included data from 164 patients with ILDs (derivation ILD cohort—USF/TGH: 118, validation IPF cohort—CC: 46). Median age (95% CI) was 69.0 (65.9–71.0) in USF/TGH cohort and 63.0 (60.0–66.0) in CC cohort. Patients in the USF/TGH cohort had a mean Forced Vital Capacity (FVC) of 62.4 ± 20.0 % predicted and a mean diffusing capacity for carbon monoxide (DLCO) of 32.6 ± 14.7 % predicted. Patients in the CC cohort had an FVC of 46.7 ± 13.8 % predicted and a DLCO of 25.6 ± 8.0 % predicted. Median mPAP in the USF/TGH cohort was 25.0 (23.0–27.0) mmHg, whereas median mPAP (95%CI) in the CC cohort was 20.5 (18.0–27.2) mmHg. Further characteristics of patients in both cohorts are presented in Table 1. Diagnosis, radiographic patterns, baseline laboratory and treatment data for patients in the USF/TGH cohort are presented in Tables 2–4. The entirety of patients from the CC cohort had IPF.

**TABLE 1:** Demographics and characteristics of patients.

Characteristics	Tampa General Hospital/University of South Florida cohort (N, %)	Cleveland clinic cohort (N, %)
Number of patients	118	46
Female gender	65 (54.6%)	17 (37%)
Age at diagnosis of ILD (years, median, 95% CI)	69.0 (65.9–71.0)	63.0 (60.0–66.0)
PH	80 (67.8%)	28 (60.9%)
6 MWD (median, 95% CI)	274.0 (257.8–289.8)	319.5 (276.2–347.2)
FVC% pred (mean ± SD)	62.4 ± 20.0	46.7 ± 13.8
DLCO% (mean ± SD)	32.6 ± 14.7	25.6 ± 8.0
mPAP (mmHg, median, 95% CI)	25.0 (23.0–27.0)	20.5 (18.0–27.2)
PCWP (mmHg, median, 95% CI)	8.0 (7.0–9.1)	7.5 (6.0–9.0)
PVR (WU, median, 95% CI)	3.6 (3.0–4.3)	3.1 (2.2–3.4)
PHOS-ILD score (median, 95% CI)	16.0 (14.9–17.1)	14.0 (12.0–17.0)

DLCO, diffusing capacity of the lung for carbon monoxide; FVC, forced vital capacity; mPAP, mean pulmonary arterial pressure; PCWP, pulmonary capillary wedge pressure; PH, pulmonary hypertension; PVR, pulmonary vascular resistance; WU, wood units; 6 MWD, 6-min walk distance.

**TABLE 2:** Diagnosis of patients from Tampa General Hospital/University of South Florida cohort.

Diagnosis	Tampa General Hospital/University of South Florida (N, %)
Number of patients	118
CTD-ILD	28 (23.7%)
IPF	25 (21.2%)
Fibrotic HP	14 (11.9%)
Non-fibrotic HP	7 (5.9%)
CPFE	7 (5.9%)
IPAF	7 (5.9%)
Idiopathic NSIP	6 (5.1%)
Unclassifiable ILD	6 (5.1%)
Familial pulmonary fibrosis	3 (2.5%)
PPFE	2 (1.7%)
Other ILDs	13 (11.0%)

CPFE, combined pulmonary fibrosis and emphysema; CTD-ILD, connective tissue disease-interstitial lung disease; HP, hypersensitivity pneumonitis; ILD, interstitial lung disease; IPAF, interstitial pneumonia with autoimmune features; IPF, idiopathic pulmonary fibrosis; NSIP, non-specific interstitial pneumonia; PPFE, pleuroparenchymal fibroelastosis.

**TABLE 3:** Radiographic patterns in Tampa General Hospital/University of South Florida cohort.

Radiographic pattern	Tampa General Hospital/University of South Florida (N, %)
Number of patients	118
Definite UIP	29 (24.6%)
Probable UIP	4 (3.4%)
Indeterminate for UIP	14 (11.9%)
Fibrotic BIP	25 (21.2%)
Non-fibrotic BIP	14 (11.9%)
CPFE	7 (5.9%)
NSIP	14 (11.9%)
PPFE	2 (1.7%)
Other	9 (7.6%)

BIP, bronchiolocentric interstitial pneumonia; CPFE, combined pulmonary fibrosis and emphysema; NSIP, non specific interstitial pneumonia; PPFE, pleuroparenchymal fibroelastosis; UIP, usual interstitial pneumonia.

**TABLE 4:** Baseline treatment and laboratory data in Tampa General Hospital/University of South Florida cohort.

Baseline data	Tampa General Hospital/University of South Florida (N, %)
Number of patients	118
Nintedanib	60 (50.8%)
Mycophenolate mofetil	40 (33.9%)
Furosemide	38 (32.2%)
Corticosteroids	28 (23.7%)
LTOT	28 (23.7%)
Pirfenidone	13 (11.0%)
BNP (pg/mL, mean ± SD)	174.4 ± 77.3
Hb (g/dL, mean ± SD)	14.3 ± 3.5
Creatinine (mg/dL, mean ± SD)	0.9 ± 0.5
Albumin (g/dL, mean ± SD)	4 ± 0.4
CRP (mg/dL, mean ± SD)	0.25 ± 0.35

BNP, B-type natriuretic peptide; CRP, C-reactive protein; Hb, hemoglobin; LTOT, long-term oxygen therapy; SD, standard deviation.

### Generation of the PHOS-ILD score

In the USF/TGH-derivation cohort, we calculated odds ratios and optimal cut-offs for the presence of PH across multiple parameters, by comparing patients with ILD-PH to those with ILD without PH. Parameters significantly associated with precapillary PH in the derivation cohort were: age > 42 years, heart rate recovery at 1 min (HRR) ≤ 15 beats per minute (bpm) in 6 MWT, 6 MWD ≤ 270 meters, B-type natriuretic peptide (BNP) > 136 pg/mL, PA/AA > 1 in HRCT, main PA diameter > 29 mm in HRCT, estimated right ventricular systolic pressure (RVSP) > 42 mmHg in echocardiogram (Table 5 and Figure 1B).

**TABLE 5:** Odds ratio for PH in Tampa General Hospital/University of South Florida cohort.

	ILD-PH	ILD without PH	OR	*P*-value
All patients	80	38	NA	NA
Age ( > 42 years of age)	77/80 (96.3%)	31/38 (81.6%)	5.80 (1.41–23.87)	**0.015**
Gender (female)	48/80 (60%)	21/38 (55.3%)	0.82 (0.38–1.80)	0.63
Race (non-white)	38/80 (47.5%)	12/38 (31.6%)	1.96 (0.87–4.42)	0.10
Syncope	7/80 (8.8%)	6/38 (15.8%)	0.51 (0.16–1.64)	0.26
LTOT	55/80 (68.8%)	27/38 (71.1%)	0.90 (0.38–2.09)	0.80
HRR ≤ 15 beats/min in 6 MWT	67/80 (83.8%)	25/38 (65.8%)	2.68 (1.09–6.56)	**0.03**
6 MWD ≤ 270 m	43/80 (53.8%)	12/38 (31.6%)	2.52 (1.12–5.68)	**0.02**
Hypoxia in 6 MWT	29/80 (36.3%)	21/38 (55.3%)	2.17 (0.99–4.76)	0.052
BNP > 136 pg/mL	26/67 (38.8%)	4/31 (12.9%)	4.09 (1.31–12.76)	**0.015**
FVC% pred/DLCO% pred > 1.8	49/80 (61.3%)	18/38 (47.4%)	1.76 (0.81–3.83)	0.15
DLCO% pred ≤ 29	34/80 (42.5%)	14/38 (36.8%)	1.27 (0.57–2.80)	0.56
PA/AA > 1 on chest CT	52/80 (65.0%)	13/38 (34.2%)	3.57 (1.58–8.05)	**0.002**
Main PA diameter > 29 mm	56/80 (70.0%)	17/38 (44.7%)	2.88 (1.30–6.40)	**0.009**
RVSP > 42 mmHg	47/80 (58.8%)	9/38 (23.7%)	4.59 (1.92–10.96)	**0.0006**

BNP, B-type natriuretic peptide; DLCO, diffusing capacity of the lung for carbon monoxide; FVC, forced vital capacity; HRR, heart rate recovery; LTOT, long term oxygen therapy; PA/AA, pulmonary artery to ascending aorta diameter ratio; OR, odds ratio; RVSP, right ventricular systolic pressure; 6 MWD; 6-min walk distance; 6 MWT, 6-min walk test. Bold values indicate statistical significance.

A PH detection model system using these parameters was developed. In particular, point values were assigned by rounding the odds ratios associated with each significant predictor of PH. We assigned 6 points if age > 42 years, 3 points if HRR ≤ 15 bpm in 6 MWT, 3 points if 6 MWD ≤ 270 meters, 4 points if BNP > 136 pg/mL, 4 points if PA/AA > 1 on HRCT, 3 points if main PA diameter > 29 mm and 5 points if estimated RVSP > 42 mmHg. Thus, the range of the PHOS-ILD score was 0–28 and ROC identified an optimal cut-off threshold ( > 14) with 80% sensitivity, 68% specificity, and an AUC: 0.78 for the detection of PH.

We also generated a simplified PH detection model using the minimum number of parameters required to achieve a similar AUC. Interestingly, exclusion of BNP did not reduce the AUC. Main PA diameter > 29 mm was removed from the simplified PHOS-ILD score following multicollinearity assessment. Main PA diameter > 29 mm demonstrated substantial multicollinearity with the PA/AA > 1, while PA/AA > 1 exhibited significance even in the multivariable logistic regression (Tables 6, 7). The range of simplified PHOS-ILD score was between 0 and 21. ROC demonstrated an optimal cut-off threshold > 12 with 76% sensitivity and 66% specificity, and an AUC of 0.78 for the detection of PH in the derivation cohort. The optimism-corrected AUC following 2,000 bootstrap resamples was 0.78, while the 10-fold cross-validated AUC was 0.77. Cross-validated calibration demonstrated an intercept of 0.04 and a slope of 0.94, with

**TABLE 6:** Assessment of multicollinearity using variance inflation factors (VIFs).

Parameters	VIF
Age ( > 42 years of age)	5.45
HRR ≤ 15 beats/min in 6 MWT	3.87
6 MWD ≤ 270 m	1.94
RVSP > 42 mmHg	2.29
PA/AA > 1 on chest CT	11.61
Main PA diameter > 29 mm	13.26
BNP > 136 pg/mL	1.73

BNP, B-$; HRR, heart rate recovery; PA/AA, pulmonary artery to ascending aorta diameter ratio; RVSP, right ventricular systolic pressure; VIF, variance inflation factors; 6 MWD, 6-min walk distance; 6 MWT, 6-min walk test.

**TABLE 7:** Multivariable logistic regression of the parameters included in the simplified PHOS-ILD score.

Variable	Coefficient	Std. error	Wald	*P*-value
Age ( > 42 years of age)	1.26016	0.86715	2.1119	0.1462
HRR ≤ 15 beats/min in 6 MWT	0.62874	0.52878	1.4138	0.2344
6 MWD ≤ 270 m	0.49550	0.48229	1.0555	0.3042
PA/AA > 1 onchest CT	0.98603	0.46926	4.4152	**0.0356**
RVSP > 42 mmHg	1.21187	0.50198	5.8281	**0.0158**
Constant	−2.03243	0.95059	4.5713	0.0325

HRR, heart rate recovery; PA/AA, pulmonary artery to ascending aorta diameter ratio; RVSP, right ventricular systolic pressure; 6 MWD, 6-min walk distance; 6 MWT, 6-min walk test. Bold values indicate statistical significance.

a Brier score of 0.17. Of note, in the subgroup analysis, PHOS-ILD score exhibited 80% sensitivity and 80% specificity (optimal threshold > 14, AUC 0.78) for PH detection in Hypersensitivity Pneumonitis, while in terms of PH detection in connective tissue disease (CTD)-ILD sensitivity was 89% and specificity 86% (optimal threshold > 14, AUC 0.96) (Table 8). In the validation cohort, the prespecified cut-off threshold of > 12, derived from the derivation cohort, yielded a sensitivity of 70% and a specificity of 70%, with an AUC of 0.74. An exploratory analysis identified > 11 as the optimal cohort-specific threshold, which yielded a sensitivity of 82% and a specificity of 65% (Table 8). Removal of further parameters worsened AUC, thus no further parameters could be removed from the simplified version.

**TABLE 8:** PHOS-ILD score was generated using the parameters that were significantly associated with precapillary PH in the derivation cohort.

	Extended PHOS-ILD score	Simplified PHOS-ILD score		
Parameters	Age > 42 years →6 points, HRR ≤ 15 beats/min in 6 MWT → 3 points, 6 MWD ≤ 270 meters → 3 points, BNP > 136 pg/mL → 4 points, PA/AA > 1 on chest CT → 4 points, main PA diameter > 29 mm→ 3 points, RVSP > 42 mmHg→ 5 points	Age > 42 years →6 points, HRR ≤ 15 beats/min in 6 MWT → 3 points, 6 MWD ≤ 270 meters → 3 points, PA/AA > 1 on chest CT → 4 points, RVSP > 42 mmHg→ 5 points		
	**Derivation cohort**	**Derivation cohort**		**Validation cohort**
Range	0–28	0–21		0–21
Derivation cohort derived optimal cut-off threshold	> 14	>12		> 12
Sensitivity	80%	76%		70%
Specificity	68%	66%		70%
AUC	0.78	0.78		0.74
		**Subgroup analysis**		**Exploratory analysis**
		**HP**	**CTD-ILD**	
Optimal cut-off threshold		>14	> 9	>11
Sensitivity		80%	89%	82%
Specificity		80%	86%	65%
AUC		0.78	0.96	0.74

Point values were assigned by rounding the odds ratios associated with each significant predictor identified. Subsequently, a simplified final version was generated using the minimum number of parameters required to achieve the same AUC. Sensitivity, specificity and AUC for PH detection in patients with ILD are presented both for the extended and the final version of PHOS-ILD score are presented in the table. AUC, area under the curve; BNP, B-type natriuretic peptide; CT, computed tomography; HRR, heart rate recovery at 1 min; PA/AA, pulmonary artery to ascending aorta diameter ratio; RVSP, right ventricular systolic pressure; 6 MWD, 6-min walk distance; 6 MWT, 6-min walk test.

### Correlation of PHOS-ILD score with parameters indicative of PH diagnosis in RHC—validation in an independent cohort

PHOS-ILD score was correlated with mPAP both in the USF/TGH cohort (rho = 0.47, *p* < 0.0001) and in the CC cohort (rho = 0.36, *p* = 0.013) (Figures 2A,B). PHOS-ILD score was also correlated with PVR in both the USF/TGH (rho = 0.48, *p* < 0.0001) and CC cohorts (rho = 0.34, *p* = 0.019) (Figures 2C,D).

**FIGURE 2:**
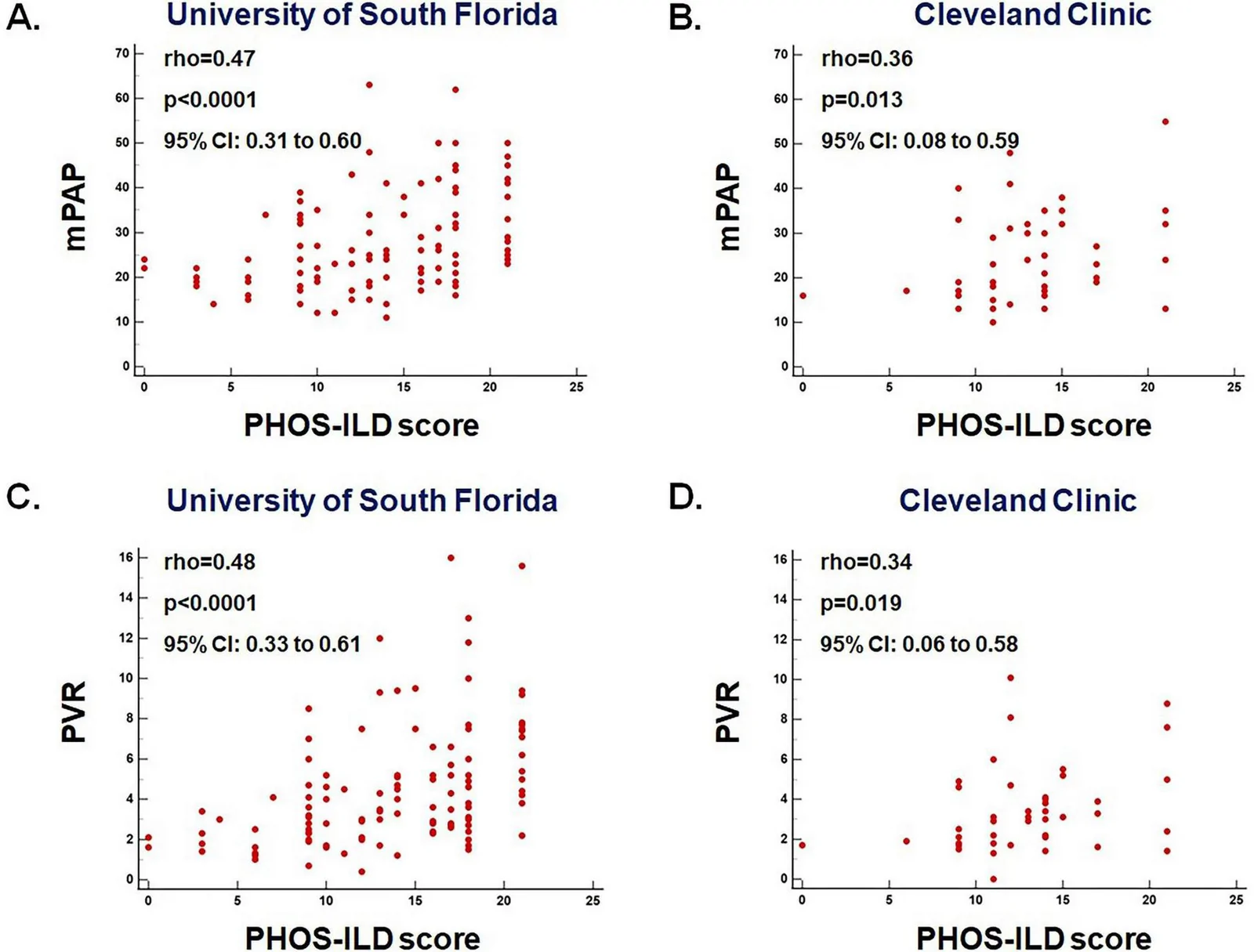
Spearman’s coefficient of rank correlation (rho) between PHOS-ILD score and mPAP in USF/TGH **(A)** and Cleveland Clinic **(B)**. Accordingly, Spearman’s coefficient of rank correlation (rho) between PHOS-ILD score and PVR in USF/TGH **(C)** and Cleveland Clinic **(D)**.

## Discussion

This retrospective, multicenter study identified key variables that predict PH in patients with ILD and presented a scoring system, named PHOS-ILD score, to optimize the selection of patients with ILD undergoing RHC for confirmation of precapillary PH presence. The identification of precapillary PH in ILD patients is essential, since there is effective treatment for this subpopulation (28). In particular, we identified that age > 42 years, HRR ≤ 15 bpm in 6 MWT, 6 MWD ≤ 270 meters, BNP > 136 pg/mL, PA/AA ratio > 1 in HRCT, main PA diameter > 29 mm in HRCT and estimated RVSP > 42 mmHg on echocardiogram were significantly associated with PH in patients with ILDs. Based on these parameters, we generated two versions of the PH scoring system, one with and another without BNP and main PA diameter. Each achieved an AUC of 0.78, indicating that both versions are robust and clinically useful tools for PH detection. With regards to the simplified score, VIF analysis demonstrated substantial multicollinearity between main pulmonary artery diameter and the PA/AA ratio, and consequently only the PA/AA ratio was retained. Removal of BNP did not worsen the AUC of the score. Thus, these two parameters were removed from the final score. Importantly, weighting variables according to the adjusted regression coefficients did not improve discriminatory performance and resulted in a less practical scoring system. In contrast, weighting predictors according to the magnitude of their univariable odds ratios yielded a simpler score with superior discrimination while maintaining ease of clinical use. Thus, point values were assigned by rounding the odds ratios associated with each significant predictor identified in the univariate analysis. Furthermore, PHOS-ILD score was associated with parameters of PH diagnosis in RHC and this was validated in an external, independent IPF cohort. Therefore, this PH detection model may pave the way to structured screening strategies and contribute to the timely detection of PH in patients with various ILDs.

Our model extends prior efforts to recognize PH in ILD through non-invasive parameters, demonstrating strong discriminative performance and reproducibility both in the overall ILD population and specifically in patients with IPF (31). In the context of previously developed models in ILDs, the PH-ILD detection tool has shown high and reproducible discriminative performance by integrating history, physical exam, 6 MWT, DLCO, HRCT of the chest and cardiac biomarkers (38, 39). Within IPF, a validated model for PH detection is the FORD model which includes four variables: FVC/DLCO ratio (F), oxygen saturation nadir during 6 MWT (O), race (R), and distance ambulated during 6 MWT (D). The FORD model demonstrated good discriminative performance in both derivation (AUC: 0.75) and validation (AUC: 0.69) cohorts (36). Most recently, FORD has been used in non-IPF ILDs, as well (40). Other models have also been proposed (4, 31, 41), although some lack external validation. Differences in discriminative performance of the PH detection models may reflect variations in cohort characteristics or variable selection.

Our model includes demographic, functional, imaging and echocardiographic parameters with an established role in PH detection. Some of these parameters are tied to abnormalities in pulmonary vasculature, while others (i.e., 6 MWD) could be identified as a result of abnormalities both in pulmonary vasculature and in pulmonary parenchyma. In terms of demographics, advanced age is a well-recognized risk factor for PH development. Multiple registries have demonstrated higher prevalence of PH with increasing age, with the highest being in subjects > 75 years of age (42). Aging promotes a vascular milieu that favors pulmonary vascular remodeling through cumulative endothelial injury, cellular senescence in endothelial and smooth muscle cells, impaired nitric oxide–dependent vasodilation and increased oxidative stress; leading to intimal thickening, medial hypertrophy and increased pulmonary vascular resistance (43, 44). These age-related maladaptive processes are enhanced in patients with ILD, as repetitive epithelial injury, hypoxemia and recurrent microvascular loss accelerate pulmonary vascular remodeling and progression to PH (25).

In the context of functional indices, we identified HRR ≤ 15 bpm in 6 MWT and 6 MWD ≤ 270 m as indicators of PH in patients with ILD. Determination obtained during the 6 MWT provide clinically useful, noninvasive indicators of PH in ILD (36, 45–47). Several studies have reported reduced 6 MWD in patients with ILD-PH compared to patients with ILD alone. Importantly, lower 6 MWD was associated with higher mPAP, worse right-ventricular systolic function, disease severity and increased mortality in patients with ILD (8, 36, 45, 46, 48). The 6 MWT informs on a variety of processes such as pulmonary-gas exchange capacity, ventilatory mechanics, peripheral muscle function and cardiac output reserve, while blunted HRR after 6 MWT indicates persistent sympathetic activation, impaired chronotropic competence and reduced stroke-volume reserve (49).

We noted a trend, but not statistical significance, for the ratio FVC% pred./DLCO% pred., which is a functional marker for PH that has been suggested by a few studies in patients with ILDs (4). Suggested thresholds of FVC% pred./DLCO% pred. that could serve as indicators of PH range from 1.3 to 1.7 in patients with ILDs (4, 46), whereas multiple expert reviews and scleroderma screening algorithms have proposed a ratio of FVC% pred./DLCO% pred. > 1.6 (50).

With regards to imaging biomarkers, we demonstrated that PA/AA > 1 in HRCT and main PA diameter > 29 mm in HRCT were significant indicators of PH in patients with ILD. This observation is in line with existing literature. Both PA/AA > 1 and main PA diameter > 29 mm have been repeatedly associated with increased mPAP and have been used as non-invasive screening markers for PH in ILDs (51, 52). Previous studies established an empiric upper normal main PA diameter of ≈ 29 mm. However, PA/AA > 1 seems to increase specificity for the detection of pulmonary vascular disease, since it controls for interindividual differences in vascular size, identifying disproportionate pulmonary arterial dilation (51). In our analysis main PA diameter exhibited substantial multicollinearity with PA/AA > 1, thus it was excluded from the simplified score (51).

PHOS-ILD score included also RVSP which is most used non-invasive tool to screen for PH. Estimated RVSP thresholds of 40–45 mmHg have been commonly employed to identify patients at risk for PH. In multiple ILD or IPF cohorts, elevated RVSP correlated with invasive hemodynamic evidence of PH, poor functional status and worse survival (35, 53–56). A major limitation of RVSP is the diagnostic performance in patients with chronic lung diseases and particularly in patients with emphysema or Combined Pulmonary Fibrosis and Emphysema. Suboptimal acoustic windows, common in ILD patients, lead to lower sensitivity (55). In our cohort, RVSP was successfully estimated in all patients; therefore, there were no missing or non-estimable RVSP values and no imputation or sensitivity analyses were required. We believe this reflects the highly selected nature of our cohort, comprising patients with ILD who underwent RHC because PH was clinically suspected. Consequently, many patients had sufficient tricuspid regurgitation to allow reliable Doppler estimation of RVSP. Nevertheless, the PHOS-ILD score was intentionally designed so that patients may still reach the diagnostic cut-off for PH even when RVSP cannot be estimated. This represents an important strength of the score, as RVSP might be unobtainable in routine echocardiographic practice, particularly in patients with ILD.

Finally, BNP, as N-terminal-pro-B-type natriuretic peptide (NT-proBNP) was not available at our center, was also identified as a significant predictor of PH in our cohort; yet, our score maintained a similar AUC even without its use. Several studies have assessed BNP or its analog NT-proBNP as a screening biomarker of PH in patients with ILD. Elevated BNP has been associated with increased systolic PAP, mPAP ≥ 25 mmHg, right ventricular dysfunction and reduced Tricuspid Annular Plane Systolic Excursion (TAPSE) (57). On the contrary, BNP lacks specificity, given that increased values may arise from left-heart disease or other causes of ventricular strain (41). Of note, BNP or NT-pro BNP determinations are not interchangeable, and not routinely done by all centers as part of the ILD workout (41).

Another important attribute of this work is that PHOS-ILD demonstrated potential utility both across a broad ILD population and within a pure IPF cohort. Validation in IPF was of particular interest, since the group of patients with Idiopathic Interstitial Pneumonias and PH seemed to have considerable benefit from inhaled treprostinil in the subgroup analysis of the INCREASE study (28). Notably, patients with IPF also experienced benefit from inhaled treprostinil in the recently published TETON studies (29, 30). In fact, the development of pulmonary vascular disease in ILD is increasingly recognized as a progressive continuum, encompassing several phenotypes: absence of PH, elevated pulmonary vascular resistance without overt PH, exercise-induced PH and finally established PH at rest (58). PHOS-ILD focuses on the identification of the later group as there are treatments available. Future studies should address whether baseline or longitudinal assessment of PHOS-ILD could enable earlier identification of ILD patients at risk of progression to precapillary PH.

Identification and detailed characterization of PH are of paramount importance and more timely than ever. In an era that multiple compounds will be available for implementation in patients with lung fibrosis and concomitant implementation of all compounds to all patients will be, at least logistically, challenging (29, 30, 59, 60), presence of PH could be an extra feature in terms of inhaled treprostinil selection. Detailed characterization of PH could also contribute to the identification of the subgroup of patients with disproportionate PH relative to the severity of the underlying ILD. Such patients may experience benefit from the addition of other compounds to inhaled treprostinil, such as sildenafil or maybe in the future from other drugs that hold promise (61–63). Finally, presence of PH could guide decisions in terms of lung transplantation (10).

Our study has also some limitations that warrant consideration. First, this study has the inherent weaknesses of a retrospective study including missing data, i.e., for BNP which is not part of the regular workout in all patients with ILDs. Second, we did not study combined pre and post capillary PH. Third, we did not quantify abnormalities of pulmonary parenchyma. Fourth, the sample size and the patient population might not fully represent all ILD subtypes or all ethnic groups. We validated our findings in an independent IPF cohort, but not in cohorts with other fibrotic ILDs. We need to acknowledge that our study comprised patients with ILD who underwent RHC because PH was clinically suspected, reflecting the intended clinical application of the PHOS-ILD score. Accordingly, the score should be considered a risk stratification tool to support referral for invasive haemodynamic assessment of patients with ILD and suspected PH rather than a population-wide screening tool for all patients with ILD. Although our design may limit generalizability to unselected ILD populations, it provides a greater number of outcome events, allowing more precise estimation of predictor effects, reduction of the risk of unstable estimates and more robust model development. A similar derivation strategy was adopted in the large, prospective, multicenter US PHINDER study, where the prediction model was developed in patients undergoing evaluation for suspected PH (64). Such approaches are gaining increasing attention, as studies conducted in unselected ILD populations have lower event rates and therefore require considerably larger sample sizes for robust model development. To further validate our findings, we performed bootstrap internal validation, 10-fold cross-validation and external validation in an independent IPF cohort. Nevertheless, as with any prediction model derived from retrospective data, some degree of optimism in the apparent performance of the derivation cohort cannot be completely excluded despite internal validation. Further external validation in independent and diverse ILD cohorts will be important to strengthen the evidence supporting the generalizability and clinical utility of the PHOS-ILD score. Finally, approximately one-quarter of our cohort comprised patients with CTD-ILD. Although such patients may exhibit overlapping Group 1 PAH and Group 3 PH phenotypes, distinguishing these entities can be challenging in routine clinical practice. In our cohort, the mean FVC was 62.4 ± 20.0%, suggesting that the majority of patients had clinically significant parenchymal lung involvement. Nevertheless, the PHOS-ILD score should be interpreted as a pragmatic risk stratification tool for patients with ILD in whom PH is suspected, rather than as a tool to differentiate the underlying PH phenotype.

Collectively, this study demonstrated that incorporation of demographic, functional, imaging, and echocardiographic parameters can enhance the early identification of precapillary PH in patients with ILD, paving the way to structured screening strategies. Incorporation of these parameters led to the PHOS-ILD score, which showed potential in terms of PH detection. Optimal PH screening through validated detection models could be crucial for the timely detection and management of patients with ILD-PH.

## Data Availability

The raw data supporting the conclusions of this article will be made available by the authors, without undue reservation.

## References

[1] Ryerson CJ Adegunsoye A Piciucchi S Hariri LP Khor YH Wijsenbeek MS et al. Update of the international multidisciplinary classification of the interstitial pneumonias: an ERS/ATS statement. *Eur Respir J.* (2025) 66:2500158. 10.1183/13993003.00158-2025 40774805

[2] Karampitsakos T Juan-Guardela BM Tzouvelekis A Herazo-Maya JD. Precision medicine advances in idiopathic pulmonary fibrosis. *EBioMedicine.* (2023) 95:104766. 10.1016/j.ebiom.2023.104766 37625268 PMC10469771

[3] Karampitsakos T Spagnolo P Mogulkoc N Wuyts WA Tomassetti S Bendstrup E et al. Lung cancer in patients with idiopathic pulmonary fibrosis: a retrospective multicentre study in Europe. *Respirology.* (2023) 28:56–65. 10.1111/resp.14363 36117239

[4] Arslan A Smith J Qureshi MR Uysal A Patel KK Herazo-Maya JD et al. Evolution of pulmonary hypertension in interstitial lung disease: a journey through past, present, and future. *Front Med.* (2023) 10:1306032. 10.3389/fmed.2023.1306032 38298504 PMC10827954

[5] Tonelli AR Sahay S. A twist in the fibrotic tale: the overlooked vasculopathy in idiopathic pulmonary fibrosis. *Am J Respir Crit Care Med.* (2025) 211:897–8. 10.1164/rccm.202502-0465ED 40208255 PMC12175955

[6] Kacprzak A Tomkowski W Szturmowicz M. Pulmonary hypertension in the course of interstitial lung diseases-a personalised approach is needed to identify a dominant cause and provide an effective therapy. *Diagnostics.* (2023) 13:2354. 10.3390/diagnostics13142354 37510098 PMC10378268

[7] Tyagi R Kant S Verma SK Kushwaha RAS Kumar S Garg R et al. Spectrum of interstitial lung diseases and their association with pulmonary hypertension. *Monaldi Arch Chest Dis.* (2021) 92:2021. 10.4081/monaldi.2021.2027 34873902

[8] Karampitsakos T Tzouvelekis A Chrysikos S Bouros D Tsangaris I Fares WH. Pulmonary hypertension in patients with interstitial lung disease. *Pulm Pharmacol Ther.* (2018) 50:38–46. 10.1016/j.pupt.2018.03.002 29605286

[9] Oliveira RK Pereira CA Ramos RP Ferreira EV Messina CM Kuranishi LT et al. A haemodynamic study of pulmonary hypertension in chronic hypersensitivity pneumonitis. *Eur Respir J.* (2014) 44:415–24. 10.1183/09031936.00010414 24743965

[10] Karampitsakos T Zweers B Hammonds J Qureshi MR Arce Guzman C Bandyopadhyay D et al. Pre-lung transplant monocyte counts are associated with post-lung transplant survival in idiopathic pulmonary fibrosis. *Am J Respir Crit Care Med.* (2026) 212:1041–5. 10.1093/ajrccm/aamag055 41738233 PMC13160946

[11] Olsson KM Corte TJ Kamp JC Montani D Nathan SD Neubert L et al. Pulmonary hypertension associated with lung disease: new insights into pathomechanisms, diagnosis, and management. *Lancet Respir Med.* (2023) 11:820–35. 10.1016/S2213-2600(23)00259-X 37591300

[12] Raghu G Nathan SD Behr J Brown KK Egan JJ Kawut SM et al. Pulmonary hypertension in idiopathic pulmonary fibrosis with mild-to-moderate restriction. *Eur Respir J.* (2015) 46:1370–7. 10.1183/13993003.01537-2014 26250495

[13] Guenther A Krauss E Tello S Wagner J Paul B Kuhn S et al. The European IPF registry (eurIPFreg): baseline characteristics and survival of patients with idiopathic pulmonary fibrosis. *Respir Res.* (2018) 19:141. 10.1186/s12931-018-0845-5 30055613 PMC6064050

[14] Nathan SD Barnett SD King CS Provencher S Barbera JA Pastre J et al. Impact of the new definition for pulmonary hypertension in patients with lung disease: an analysis of the United Network for Organ Sharing database. *Pulm Circ.* (2021) 11:2045894021999960. 10.1177/2045894021999960 33868639 PMC8020109

[15] Karampitsakos T Tourki B Herazo-Maya JD. The Dawn of Precision Medicine in Fibrotic Interstitial Lung Disease. *Chest.* (2025) 167:1120–32. 10.1016/j.chest.2024.10.042 39521375 PMC12001815

[16] Humbert M Kovacs G Hoeper MM Badagliacca R Berger RMF Brida M et al. 2022 ESC/ERS Guidelines for the diagnosis and treatment of pulmonary hypertension. *Eur Heart J.* (2022) 43:3618–731. 10.1093/eurheartj/ehac237 36017548

[17] Shlobin OA Adir Y Barbera JA Cottin V Harari S Jutant EM et al. Pulmonary hypertension associated with lung diseases. *Eur Respir J.* (2024) 64:2401200. 10.1183/13993003.01200-2024 39209469 PMC11525344

[18] Kovacs G Bartolome S Denton CP Gatzoulis MA Gu S Khanna D et al. Definition, classification and diagnosis of pulmonary hypertension. *Eur Respir J.* (2024) 64:2401324. 10.1183/13993003.01324-2024 39209475 PMC11533989

[19] Dhont S Zwaenepoel B Vandecasteele E Brusselle G De Pauw M. Pulmonary hypertension in interstitial lung disease: an area of unmet clinical need. *ERJ Open Res.* (2022) 8:272–2022. 10.1183/23120541.00272-2022 36382238 PMC9661248

[20] Lawrence A Myall KJ Mukherjee B Marino P. Converging pathways: a review of pulmonary hypertension in interstitial lung disease. *Life.* (2024) 14:1203. 10.3390/life14091203 39337985 PMC11433497

[21] Nathan SD Hassoun PM. Pulmonary hypertension due to lung disease and/or hypoxia. *Clin Chest Med.* (2013) 34:695–705. 10.1016/j.ccm.2013.08.004 24267299

[22] Nathan SD Shlobin OA Ahmad S Koch J Barnett SD Ad N et al. Serial development of pulmonary hypertension in patients with idiopathic pulmonary fibrosis. *Respiration.* (2008) 76:288–94. 10.1159/000114246 18216461

[23] Nikkho SM Richter MJ Shen E Abman SH Antoniou K Chung J et al. Clinical significance of pulmonary hypertension in interstitial lung disease: a consensus statement from the Pulmonary Vascular Research Institute’s innovative drug development initiative-Group 3 pulmonary hypertension. *Pulm Circ.* (2022) 12:e12127. 10.1002/pul2.12127 36016668 PMC9395696

[24] Grünig E Huscher D Pittrow D Vizza D Hoeper MM. Pulmonary hypertension due to lung disease – Results from COMPERA. *Eur Respir J.* (2015) 46:OA5000. 10.1183/13993003.congress-2015.OA5000 34385266

[25] Jose A Sood N Elwing JM Akkanti B Bajwa A Bernardo R et al. Pulmonary Hypertension Associated With Interstitial Lung Diseases. *Chest.* (2025) 169:220–9. 10.1016/j.chest.2025.07.4107 40939937 PMC12895325

[26] Karyofyllis P Demerouti E Habibis P Apostolopoulou S Tsetika EG Tsiapras D. Should we change the target of therapy in pulmonary hypertension? *Life.* (2023) 13:1202. 10.3390/life13051202 37240847 PMC10221333

[27] Burger CD Ghandour M Padmanabhan Menon D Helmi H Benza RL. Early intervention in the management of pulmonary arterial hypertension: clinical and economic outcomes. *Clin Outcomes Res.* (2017) 9:731–9. 10.2147/CEOR.S119117 29200882 PMC5703162

[28] Waxman A Restrepo-Jaramillo R Thenappan T Ravichandran A Engel P Bajwa A et al. Inhaled treprostinil in pulmonary hypertension due to interstitial lung disease. *N Engl J Med.* (2021) 384:325–34. 10.1056/NEJMoa2008470 33440084

[29] Nathan SD Smith P Deng C De Salvo M Wuyts W Pavie-Gallegos J et al. Inhaled treprostinil for idiopathic pulmonary fibrosis. *N Engl J Med.* (2026) 395:127–37. 10.1056/NEJMoa2512911 41812190

[30] Nathan SD Smith P Deng C King CS De Salvo M Weigt SS et al. Phase 3 trials of inhaled treprostinil for idiopathic pulmonary fibrosis. *N Engl J Med.* (2026) 395:115–26. 10.1056/NEJMoa2501488 42149993

[31] Vitulo P Piccari L Wort SJ Shlobin OA Kovacs G Vizza CD et al. Screening and diagnosis of pulmonary hypertension associated with chronic lung disease (PH-CLD): a consensus statement from the pulmonary vascular research institute’s innovative drug development initiative-group 3 pulmonary hypertension. *Pulm Circ.* (2024) 14:e70005. 10.1002/pul2.70005 39659477 PMC11629413

[32] Humbert M Kovacs G Hoeper MM Badagliacca R Berger RMF Brida M et al. 2022 ESC/ERS Guidelines for the diagnosis and treatment of pulmonary hypertension. *Eur Respir J.* (2023) 61:2200879. 10.1183/13993003.00879-2022 36028254

[33] Raghu G Remy-Jardin M Richeldi L Thomson CC Inoue Y Johkoh T et al. Idiopathic pulmonary fibrosis (an Update) and progressive pulmonary fibrosis in adults: an official ATS/ERS/JRS/ALAT clinical practice guideline. *Am J Respir Crit Care Med.* (2022) 205:e18–47. 10.1164/rccm.202202-0399ST 35486072 PMC9851481

[34] Travis WD Costabel U Hansell DM King TE Lynch DA Nicholson AG et al. An official American Thoracic Society/European Respiratory Society statement: update of the international multidisciplinary classification of the idiopathic interstitial pneumonias. *Am J Respir Crit Care Med.* (2013) 188:733–48. 10.1164/rccm.201308-1483ST 24032382 PMC5803655

[35] Rahaghi FF Kolaitis NA Adegunsoye A de Andrade JA Flaherty KR Lancaster LH et al. Screening strategies for pulmonary hypertension in patients with interstitial lung disease: a multidisciplinary delphi study. *Chest.* (2022) 162:145–55. 10.1016/j.chest.2022.02.012 35176276 PMC9993339

[36] Nathan SD Chandel A Wang Y Xu J Shao L Watkins TR et al. Derivation and validation of a noninvasive prediction tool to identify pulmonary hypertension in patients with IPF: evolution of the model FORD. *J Heart Lung Transplant.* (2024) 43:547–53. 10.1016/j.healun.2023.11.005 37979926

[37] Coghlan JG Denton CP Grünig E Bonderman D Distler O Khanna D et al. Evidence-based detection of pulmonary arterial hypertension in systemic sclerosis: the DETECT study. *Ann Rheum Dis.* (2014) 73:1340–9. 10.1136/annrheumdis-2013-203301 23687283 PMC4078756

[38] Parikh R Konstantinidis I O’Sullivan DM Farber HW. Pulmonary hypertension in patients with interstitial lung disease: a tool for early detection. *Pulm Circ.* (2022) 12:e12141. 10.1002/pul2.12141 36225536 PMC9531548

[39] Parikh R O’Sullivan DM Farber HW. The PH-ILD Detection tool: external validation and use in patients with ILD. *Pulm Circ.* (2023) 13:e12273. 10.1002/pul2.12273 37564922 PMC10410234

[40] Kim HC Chandel A King CS Kim MJ Shawabkeh M Arunachalam A et al. Performance of the FORD versus other available models for the noninvasive prediction of pulmonary hypertension in patients with interstitial lung disease. *Lung.* (2025) 203:27. 10.1007/s00408-024-00783-2 39841267 PMC11754375

[41] Arvanitaki A Diller GP Gatzoulis MA McCabe C Price LC Wort SJ. Noninvasive diagnostic modalities and prediction models for detecting pulmonary hypertension associated with interstitial lung disease: a narrative review. *Eur Respir Rev.* (2024) 33:240092. 10.1183/16000617.0092-2024 39384306 PMC11462299

[42] Örem C. Epidemiology of pulmonary hypertension in the elderly. *J Geriatr Cardiol.* (2017) 14:11–6. 10.11909/j.issn.1671-5411.2017.01.001 28270836 PMC5329727

[43] Culley MK Chan SY. Endothelial senescence: a new age in pulmonary hypertension. *Circ Res.* (2022) 130:928–41. 10.1161/CIRCRESAHA.121.319815 35298304 PMC8943914

[44] Bousseau S Sobrano Fais R Gu S Frump A Lahm T. Pathophysiology and new advances in pulmonary hypertension. *BMJ Med.* (2023) 2:e000137. 10.1136/bmjmed-2022-000137 37051026 PMC10083754

[45] Swigris JJ Olson AL Shlobin OA Ahmad S Brown KK Nathan SD. Heart rate recovery after six-minute walk test predicts pulmonary hypertension in patients with idiopathic pulmonary fibrosis. *Respirology.* (2011) 16:439–45. 10.1111/j.1440-1843.2010.01877.x 20946337 PMC3753822

[46] Joseph P Savarimuthu S Zhao J Yan X Oakland HT Cullinan M et al. Noninvasive determinants of pulmonary hypertension in interstitial lung disease. *Pulm Circ.* (2023) 13:e12197. 10.1002/pul2.12197 36814586 PMC9939578

[47] Rezende CF Mancuzo EV Corrêa RA. Heart rate recovery in 1 minute after the 6-minute walk test predicts adverse outcomes in pulmonary arterial hypertension. *PLoS One.* (2022) 17:e0268839. 10.1371/journal.pone.0268839 35622825 PMC9140226

[48] du Bois RM Albera C Bradford WZ Costabel U Leff JA Noble PW et al. 6-Minute walk distance is an independent predictor of mortality in patients with idiopathic pulmonary fibrosis. *Eur Respir J.* (2014) 43:1421–9. 10.1183/09031936.00131813 24311766

[49] Harari S Wells AU Wuyts WA Nathan SD Kirchgaessler KU Bengus M et al. The 6-min walk test as a primary end-point in interstitial lung disease. *Eur Respir Rev.* (2022) 31:220087. 10.1183/16000617.0087-2022 36002171 PMC9724818

[50] Waxman AB Elia D Adir Y Humbert M Harari S. Recent advances in the management of pulmonary hypertension with interstitial lung disease. *Eur Respir Rev.* (2022) 31:210220. 10.1183/16000617.0220-2021 35831007 PMC9724812

[51] Truong QA Massaro JM Rogers IS Mahabadi AA Kriegel MF Fox CS et al. Reference values for normal pulmonary artery dimensions by noncontrast cardiac computed tomography: the Framingham Heart Study. *Circ Cardiovasc Imaging.* (2012) 5:147–54. 10.1161/CIRCIMAGING.111.968610 22178898 PMC3275437

[52] Ratanawatkul P Oh A Richards JC Swigris JJ. Performance of pulmonary artery dimensions measured on high-resolution computed tomography scan for identifying pulmonary hypertension. *ERJ Open Res.* (2020) 6:232–2019. 10.1183/23120541.00232-2019 32055634 PMC7008140

[53] Nathan SD Shlobin OA Barnett SD Saggar R Belperio JA Ross DJ et al. Right ventricular systolic pressure by echocardiography as a predictor of pulmonary hypertension in idiopathic pulmonary fibrosis. *Respir Med.* (2008) 102:1305–10. 10.1016/j.rmed.2008.03.022 18619825 PMC2649722

[54] Huston JH Maron BA French J Huang S Thayer T Farber-Eger EH et al. Association of mild echocardiographic pulmonary hypertension with mortality and right ventricular function. *JAMA Cardiol.* (2019) 4:1112–21. 10.1001/jamacardio.2019.3345 31532457 PMC6751783

[55] Ni JR Yan PJ Liu SD Hu Y Yang KH Song B et al. Diagnostic accuracy of transthoracic echocardiography for pulmonary hypertension: a systematic review and meta-analysis. *BMJ Open.* (2019) 9:e033084. 10.1136/bmjopen-2019-033084 31871259 PMC6937087

[56] van de Veerdonk MC Vonk-Noordegraaf A Vachiery JL. Unbowed, unbent, unbroken: predicting pulmonary hypertension using echocardiography. *Eur Respir J.* (2022) 60:2200481. 10.1183/13993003.00481-2022 35926868

[57] Ruocco G Cekorja B Rottoli P Refini RM Pellegrini M Di Tommaso C et al. Role of BNP and echo measurement for pulmonary hypertension recognition in patients with interstitial lung disease: an algorithm application model. *Respir Med.* (2015) 109:406–15. 10.1016/j.rmed.2014.12.011 25613108

[58] Balakrishnan B Marakini A Detloff L Mahalwar G Lane JE Paul D et al. Invasive cardiopulmonary exercise testing identifies distinctive hemodynamic phenotypes in patients with interstitial lung disease and exercise intolerance. *Respiration.* (2026) 105:715–26. 10.1159/000550078 41397010 PMC12854684

[59] Maher TM Assassi S Azuma A Cottin V Hoffmann-Vold AM Kreuter M et al. Nerandomilast in patients with progressive pulmonary fibrosis. *N Engl J Med.* (2025) 392:2203–14. 10.1056/NEJMoa2503643 40388329

[60] Richeldi L Azuma A Cottin V Kreuter M Maher TM Martinez FJ et al. Nerandomilast in patients with idiopathic pulmonary fibrosis. *N Engl J Med.* (2025) 392:2193–202. 10.1056/NEJMoa2414108 40387033

[61] Behr J Nathan SD Wuyts WA Mogulkoc Bishop N Bouros DE Antoniou K et al. Efficacy and safety of sildenafil added to pirfenidone in patients with advanced idiopathic pulmonary fibrosis and risk of pulmonary hypertension: a double-blind, randomised, placebo-controlled, phase 2b trial. *Lancet Respir Med.* (2021) 9:85–95. 10.1016/S2213-2600(20)30356-8 32822614

[62] clinicaltrials.gov,. (2026). Available online at: https://clinicaltrials.gov/study/NCT06635850 (accessed February 2, 2026).

[63] Poor HD Zhang J Hannah-Clark S Saini S Eisenberg E Wang J et al. Sotatercept improves small airway disease and hyperinflation in patients with pulmonary hypertension. *Eur Respir J.* (2026) 67:2502731. 10.1183/13993003.02731-2025 41856569

[64] Zisman D Sahay S Bandyopadhyay D Al-Astal A Broderick M Caudell Stamper D et al. Screening for pulmonary hypertension in interstitial lung disease: preliminary results from the PHINDER study. *Adv Ther.* (2026) 43:2071–89. 10.1007/s12325-026-03508-4 41806275 PMC13156216

